# Treating children traumatized by war and Tsunami: A comparison between exposure therapy and meditation-relaxation in North-East Sri Lanka

**DOI:** 10.1186/1471-244X-9-22

**Published:** 2009-05-13

**Authors:** Claudia Catani, Mahendran Kohiladevy, Martina Ruf, Elisabeth Schauer, Thomas Elbert, Frank Neuner

**Affiliations:** 1 Department of Psychology, University of Bielefeld, 33501 Bielefeld, Germany; 2 vivo, Casella Postale no.17, Castelplanio Stazione, I-60032 Ancona, Italy; 3 Vallikamam Educational Zonal Office, Vallikamam, Sri Lanka; 4 Department of Psychology, University of Konstanz, 78457 Konstanz, Germany

## Abstract

**Background:**

The North-Eastern part of Sri Lanka had already been affected by civil war when the 2004 Tsunami wave hit the region, leading to high rates of posttraumatic stress disorder (PTSD) in children. In the acute aftermath of the Tsunami we tested the efficacy of two pragmatic short-term interventions when applied by trained local counselors.

**Methods:**

A randomized treatment comparison was implemented in a refugee camp in a severely affected community. 31 children who presented with a preliminary diagnosis of PTSD were randomly assigned either to six sessions Narrative Exposure Therapy for children (KIDNET) or six sessions of meditation-relaxation (MED-RELAX). Outcome measures included severity of PTSD symptoms, level of functioning and physical health.

**Results:**

In both treatment conditions, PTSD symptoms and impairment in functioning were significantly reduced at one month post-test and remained stable over time. At 6 months follow-up, recovery rates were 81% for the children in the KIDNET group and 71% for those in the MED-RELAX group. There was no significant difference between the two therapy groups in any outcome measure.

**Conclusion:**

As recovery rates in the treatment groups exceeded the expected rates of natural recovery, the study provides preliminary evidence for the effectiveness of NET as well as meditation-relaxation techniques when carried out by trained local counselors for the treatment of PTSD in children in the direct aftermath of mass disasters.

**Trial registration:**

ClinicalTrials.gov Identifier:NCT00820391

## Background

Mass disasters are a major challenge for child mental health care providers. Epidemiological studies have found epidemic rates of psychological disorders, particularly posttraumatic stress disorder (PTSD), in children who have been affected by war [1], natural disaster [2], and natural disaster and war combined [3,4]. As most mass disasters strike low-income countries, and because emergency situations call for a variety of humanitarian aid beyond psychological interventions, resources for the provision of mental health assistance are usually very low despite the high need. Consequently, interventions have to be tailored to the context of mass disasters. In particular, they have to be pragmatic, short, and administrable by local professionals without lengthy training or academic education in psychological or medical fields. However, since research has shown that some trauma interventions can be ineffective or even harmful, especially in the acute phase after the traumatic event [5,6], only psychological interventions with empirically verified efficacy should be applied during emergency situations.

Even though treatment outcome studies for traumatized children are still scarce compared to research on adult treatment [7], some randomized trials in industrialized countries have identified effective approaches for the treatment of children. In particular, cognitive behavioral therapy (CBT) including trauma exposure techniques has proven to be effective for child victims of sexual abuse [8-10] and other forms of violence [11,12]. The findings of a study on the effectiveness of brief trauma/grief-focused psychotherapy that has been carried out after an earthquake in Armenia [13] suggest that CBT-like methods can also be promising interventions in the context of mass disasters.

The goal of the present study was to test short-term treatments when applied by local counselors in the acute aftermath of a mass disaster, in a population already affected by prior conflicts and crisis. We chose Narrative Exposure Therapy (NET), a brief trauma-focused treatment approach developed to meet the needs of traumatized survivors of war and torture [14]. In contrast to other exposure treatments for PTSD, the patient does not identify a single traumatic event as a target in therapy. Instead, NET constructs a narrative that covers the patient's entire life, while giving a detailed account of past traumatic experiences. The efficacy of NET with adults and adolescents affected by war and torture has been proven in randomized controlled trials [15-17]. KIDNET, a version of NET adapted for the treatment of children [18], has been tested in a pilot study in an Ugandan refugee camp with Somali refugee children diagnosed with PTSD [19,20] with promising results.

As an active comparison protocol, we chose a treatment procedure that was applicable in the local context and available in the immediate aftermath of the Tsunami disaster in Sri Lanka. Meditation-relaxation techniques such as breathing exercise or mantra chanting represent exercises that are rooted in the Tamil (Hindu) culture and are well known to both children and local counselors. From a clinical point of view, some preliminary knowledge supports the feasibility of such a treatment protocol with traumatized populations. For example, meditation has been tried with Vietnam veterans [21] and war-traumatized adolescents in Kosovo [22]. Mindful meditation interventions have been suggested as a useful tool to decrease avoidance in traumatized patients [23]. These techniques aim at helping the client to increasingly focus the awareness on the present moment thereby increasing the ability to contact painful feelings, images and thoughts from the past without engaging in avoidance strategies.

The present study was carried out within the first months after the tsunami disaster in Sri Lanka. The flood wave had destroyed widespread coastal areas, especially in the east and the north of the country. In this time, the affected regions were still in an emergency condition. Officials estimated more than 30,000 causalities, and hundreds of thousands of inhabitants had to be relocated to refugee camps. In order to avoid epidemics, humanitarian assistance concentrated on providing food, water, and medical treatment. Nevertheless, the public media already reflected fears of psychological trauma, particularly among children. In fact, a study carried out by our workgroup three to four weeks after the disaster found high prevalence rates of PTSD especially in the North-Eastern coastal regions that have already been affected by two decades of civil war [4]. In response to the high rates of traumatization in children and the urgent request of targeted mental health interventions, we decided to provide immediate treatments to the most affected area at the Northern tip of the country (Manadkadu) and to evaluate the efficacy of therapies within a randomized controlled trial. This was only possible because we could build on a school-based mental health structure for war-affected children that had been established before the Tsunami. Within this program, a group of Tamil teacher counselors had already been trained in KIDNET, as well as in a standardized meditation-relaxation protocol that had been developed by local mental health experts.

The design of the study was compromised by responding to ethical concerns raised in the communities as well as by aid organizations regarding research conducted in the acute phase following a mass disaster. Unfortunately, it was not possible to have a third group of children without active treatment as a waiting list condition to control for spontaneous symptom remission. Given the massive request for trauma interventions among the Tsunami victims, we were urged to offer immediate treatment for all children diagnosed with PTSD. In addition, it was unsure whether the children in the waiting list group could be relocated at follow-up to offer them treatment after the waiting period. Furthermore, by the time this study was carried out, the whole coast line of Sri Lanka's North East was destroyed and transportation and communication were extremely difficult. An extension of the study including more participants and camps would have requested human and financial resources as well as transport and logistical solutions that were not available in this specific situation.

We had to expect that the majority of the Tsunami affected children had already been victimized by the civil war or other traumatic events [4]. In theory, a more complex traumatization involving multiple event types leads to a more severe pathology and may be more difficult to treat [18]. Nevertheless, in face of the size of the disaster, we decided to limit treatment duration to six sessions, also to make sure that therapies could be completed before the expected relocation of children to more permanent shelters. Apart from this reason, interventions tailored to the context of mass disasters such as the Tsunami should be pragmatic and short to allow for a high number of affected individuals to be treated within a short time.

In conclusion, the aim of the present study was to examine, whether highly affected children with a preliminary diagnosis of PTSD would profit more from KIDNET or from a mediation-relaxation protocol. The main outcome measure was the PTSD symptom severity score. Problems in functioning and physical health symptoms were used as secondary outcome measures. By using trained teachers as therapists, we also wanted to test whether local counselors with a specific training in trauma therapy are able to apply psychological interventions such as KIDNET and meditation-relaxation in the immediate aftermath of a mass disaster.

## Methods

### Setting

This study was conducted in response to an initial needs assessment for children affected by the tsunami in northeastern Sri Lanka within the framework of an ongoing psychosocial school program. In the immediate aftermath of the natural disaster, an epidemiological survey among children living in three severely affected coastal communities in different parts of the country had been carried out. Results of this survey [4] yielded a 45% prevalence rate of post traumatic stress disorder among children in the North-East of the country affected by the tsunami. Diagnostic interviews and subsequent therapies were conducted in two provisional refugee camps located in the village Manadkadu in the Vadamarachchi region in northern Sri Lanka. Manadkadu was completely destroyed by the tsunami. Like many other communities in the North-East of the country, the village had also been severely affected by the Sri Lankan civil war.

### Participants

**Figure 1:**
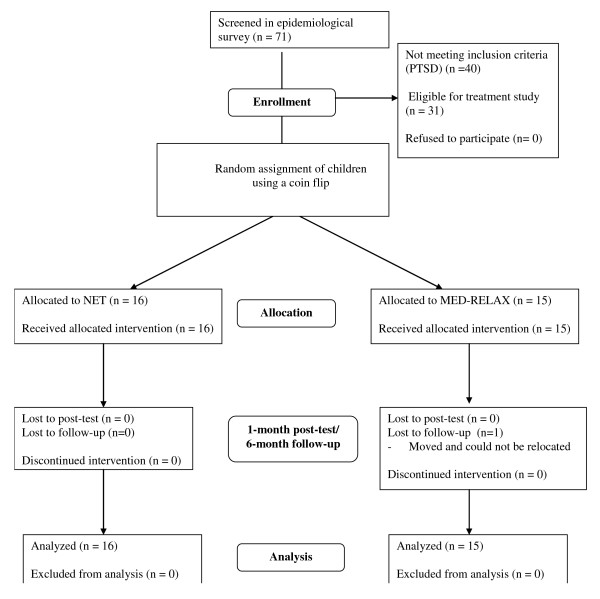
**Flowchart of study protocol**.

The population of the initial assessment consisted of children in the age range of 8 to 14 years living in the newly erected camps. All 71 eligible children who were present in the camps at the day of the interview were interviewed (see flowchart figure 1). The interviews took place three weeks after the tsunami. Consequently, the diagnosis of PTSD applied here was still tentative although a preliminary PTSD diagnosis including all DSM-IV criteria except the time criterion is a strong predictor for the development of chronic PTSD in children [24].

While no respondent met the exclusion criteria of mental retardation, psychosis or any neurological disorder, 31 children met the diagnostic criteria of preliminary PTSD and were informed about the randomized trial. All of them were willing to participate and gave their written informed consent. In addition, informed consent was obtained from one of the primary caregivers or parents of each child. The study was approved by the Ethical Review Board of the University of Konstanz and by the Ministry of Education of Sri Lanka.

Sociodemographic and clinical characteristics of the participants in the two treatment groups are shown in Table 1. As confirmed by χ^2 ^tests and t-tests, there were no systematic group differences in any of the sociodemographic variables or trauma-related characteristics, including the number of previous event types and the initial symptom score.

**Table 1:** Sociodemographic and clinical characteristics of participants in the different treatment groups.

	KIDNET (n = 16)	Meditation-Relaxation (n = 15)
Age in years: M (SD)	11.6 (2.0)	12.3 (2.0)
		
Sex: N (%)		
Male	10 (62.5)	7 (46.7)
Female	6 (37.5)	8 (53.3)
		
Years attending school: M (SD)	6.7 (2.0)	6.6 (2.0)
		
Father: N (%)		
dead	2 (12.5)	3 (20.0)
alive	14 (87.5)	12 (80.0)
		
Mother: N (%)		
dead	1 (6.3)	2 (13.3)
alive	15 (93.7)	13 (86.7)
		
No. of dead siblings: M (SD)	0.7 (1.0)	1.0 (1.2)
		
No. of physical complaints: M (SD)	1.8 (1.3)	1.8 (1.3)
		
Traumatic event types: M (SD)	4.4 (1.9)	4.7 (2.3)
		
PTSD symptom score: M (SD)	37.9 (14.8)	36.7 (14.9)
		
Worst event: N (%)		
Tsunami	14 (87.5)	11 (73.3)
other	2 (12.5)	4 (26.7)
		
Affected by traumatic war experiences:		
No	6 (37.5)	4 (26.7)
Yes	10 (62.5)	11 (73.3)

Note. χ^2 ^tests and t-tests did not show any significant group differences

### Local team of interviewers and therapists

Therapists and interviewers were recruited from a group of school teachers who had previously (prior to the Tsunami) been trained as master counselors [25] to assist children with war-related trauma (cooperation between the Zonal Department of Education Vallikamam; Shantiham counseling centre, the German Technical Cooperation – GTZ, and the NGO vivo). The master counselor training consisted of 76 training days of approximately 10 hours each, usually spreading over a total period of 6–12 months. The training included theoretical as well as practical elements. Participants attended training blocks and returned to school to practice the training contents. After completion of the formal training, counselors received supervision by the local trainers. The training curriculum was based on the "Child Mental Health" manual, which had been developed by local experts [26]. Training topics were basic counseling skills, mental health diagnosis, and trauma treatment with a focus on Narrative Exposure Therapy, as well as a meditation-relaxation protocol that had been developed by the local team of clinical experts. Counselors who were available in the immediate aftermath of the tsunami to relocate their activities to the affected regions received a 4-day refresher workshop to be prepared for the acute conditions after the tsunami. In addition, counselors selected for this study received detailed instructions on conducting the clinical questionnaires with the children, as well as information on the design and protocol of the randomized controlled treatment trial. The counselors who participated in this study had not previously worked in the schools of Manadkadu and were, therefore, not known beforehand to any of the children receiving therapies.

### Instruments

DSM-IV diagnosis and severity of PTSD were assessed with the UCLA PTSD Index for DSM-IV (UPID) [27] used in interview form. In a previous study, this instrument had been translated into Tamil following standard principles of instrument translation and validation [28]. Rather than relying on a cut-off criterion, a diagnosis of PTSD was made by corresponding the various DSM-IV criteria with the individual items of the UPID. As the UPID does not assess problems in functioning, we added a five-item scale to assess problems in functioning in different areas of children's life. Five simple questions related to problems in functioning in different areas of children's life (e.g. social relationships, family life, and general life satisfaction) were added to the interview. Impairments in psychosocial functioning were quantified as the number of "yes" answers to the five questions.

To estimate the severity of Tsunami exposure in the two post-Tsunami samples, five questions related to the Tsunami experience were added [29]. For instance, children were asked "Did you see the big wave close by?" and "Were you caught by the wave?" The five questions were answered yes or no. The score for objective Tsunami exposure was the number of "yes" answers. Physical problems were assessed using five questions about the presence of specific somatic complaints in the last four weeks. The complaints were headache, stomach ache, fever, vomiting, and diarrhea. For instance, the child was asked "Have you had diarrhea in the last four weeks?" and the item was coded "yes" or "no" according to the child's answer. The outcome measure for physical health was defined as the number of "yes" answers to the question about somatic complaints.

### Procedure

Each child who fulfilled eligibility criteria and who was willing to participate was randomly assigned (using a coin flip) either to Narrative Exposure Therapy for children (KIDNET) or meditation-relaxation. Treatments consisted of six sessions lasting 60 to 90 minutes each. In both treatment conditions, the six sessions were completed within a two weeks period. Four to five weeks after the last treatment session, post-test including the same instruments as used in the pre-test were carried out by a group of local counselors who were blind for the individual participant's treatment condition. Approximately six months after the treatment, the same group of interviewers carried out a follow-up interview with children. All interviews were supervised by the local supervisor and by one clinical psychologist from Konstanz University. Most posttests and follow-up tests were conducted in a transitory camp where most people, who initially had found shelter in the provisional camp, had moved to. For those who had moved to other places, for instance to relatives' houses, interviews were carried out at their respective places.

### Treatments

Both treatment modules consisted of six sessions lasting 60 to 90 minutes. Treatments were carried out by six female local counselors. To rule out a possible therapist effect, each therapist provided approximately the same number of treatments in both conditions. Treatment adherence and quality was monitored by requesting therapists to fill out a detailed protocol after each session. In addition, two clinical psychologists from the University of Konstanz, as well as the local clinical supervisor, carried out random observations of single therapy sessions. Throughout the entire treatment phase, regular supervision meetings were offered to the team of therapists. No major deviations from treatment protocol were detected.

#### KIDNET

During the six KIDNET sessions, the child, assisted by the therapist, constructs a detailed chronological account of his or her own biography. Particular attention is given on any traumatic experiences, including those related to the Tsunami, as well as those linked to violence and war situations. The autobiography is recorded by the therapist in written form and corrected and filled with details with each subsequent reading. Aim of this procedure is to transform the generally fragmented reports of the traumatic experiences into a coherent narrative. During the confrontation with the aversive life events, therapists ask for current and past emotional, physiological, cognitive, and behavioral reactions, and they probe for respective observations. The child is encouraged to relive these emotions while narrating. The exposure to the traumatic experience is not terminated, until the related fear reaction presented and reported by the patient does not show a significant diminution. In the last session, the participant receives a written report of his biography [14,18,30].

#### MED-RELAX (meditation-relaxation protocol)

With the help of a team of master counselors, the manual for meditation-relaxation was written by author KM, who also supervised both treatment protocols as the local senior master trainer. The first session of MED-RELAX started with psychoeducation, followed by a thorough assessment of the child's current problems, and it ended with a breathing exercise of at least 15 min. Each following session started and ended with a 15 min breathing exercise, guiding the child to achieve relaxation by attaining a conscious focus in ones mind on the incoming and outgoing breath. The middle part of the following sessions consisted of different meditation and relaxation techniques and exercises, including 'inner peace meditation' (session 2, 25 min), 'uchchadana mantra chanting' (session 3, 25 min), 'progressive muscle relaxation' (session 4, 25 min), 'ice cream body relaxation' (session 5, 25 min), and 'inner light meditation' (session 6, 25 min). Based on the detailed manual, each meditation and relaxation exercise was read out in the same way by the master counselors. For instance, the "ice cream body relaxation" was introduced in the following way in the manual: "Ice Cream Body Relaxation" (20–25 min). Have a bed sheet or mat on the floor and ask the client to stand on it with bare feet. Have relaxation music in the background and guide the client as follows:

"Stand up and raise your hands upwards. Imagine that you are an ice cream now. In the air this ice cream will melt gradually. Likewise your body also relaxes gently. (In between the music, the instructions come very gradually and slowly). The tension of your body reduces step by step and you are melting more and more. The height of this ice cream will reduce as it melts. Likewise your height goes down slowly.....slowly....some more....some more....it melts more....and comes flat to the ground. (Repeat the sentence slowly many times until the client lies flat on the floor and relaxes)."

Children were instructed to practice the meditation techniques as homework for about one hour per day.

## Results

After randomization, 16 children were offered KIDNET, and 15 children were offered the meditation-relaxation therapy. All children agreed to participate, and all of them completed the full treatment. One child moved to an unknown place and could not be located for the 6-months follow up. We planned to perform both a treatment completer, as well as an intent-to-treat analysis, using last-observation-carried forward. However, as only a single case was missing, there was no difference in results between analysis strategies. Results reported here are based on the analysis of all 31 children for the post-test, and the 30 children with complete data for the follow-up.

### PTSD Symptom Score

**Figure 2:**
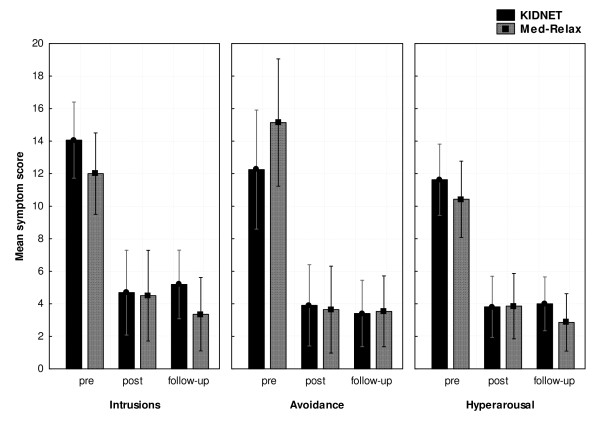
**Means of UPID subscores for the PTSD symptom clusters intrusions, avoidance, and hyperarousal at pre-treatment, posttest, and follow-up, separately for each treatment group**. Error bars indicate 95% confidence intervals.

Symptom scores across time points are presented in table 2. A repeated measures analysis of variance (ANOVA) was calculated with Time as a three-level within-subject variable and Treatment as a two-level between subjects variable for UPID total score as the main outcome measure. The ANOVA resulted in a significant main effect for the factor time (F(2,56) = 54.15; p < 0.001), whereby the Time × Treatment interaction was not significant (p = 0.9). We repeated this analysis for each of the UPID subscales intrusions, avoidance, and hyperarousal. Mean scores for the three symptom clusters at different time points are illustrated in Figure 2. No significant Time × Treatment interaction was found.

**Table 2:** Means and standard deviations of UPID symptom scores of the two treatment groups at pre-test, post-test, and follow up time points.

	pre-test	post-test	6-months follow-up
KIDNET	37.94 (14.8)	12.41 (14.15)	12.3 (10.87)
MED-RELAX	36.58 (14.9)	12.59 (11.06)	9.75 (8.63)

Note. Post-test effect sizes: 1.76 (CI 0.9–2.5) for the KIDNET group and 1.83(CI 0.9–2.6) for MED-RELAX; Follow-up effect sizes: 1.96 (CI 1.1–2.8) for KIDNET and 2.20 (CI 1.2–3.0) for MED-RELAX. No significant differences between groups were found.

Within-group effect sizes (Cohen's d) were calculated by dividing the change of mean between pre- and posttest by the pooled standard deviation of the UPID score at pre- and posttest. Effect sizes at 6-months follow-up resulted from analogous comparisons between the means at pretest and at the 6-months follow-up. Effect sizes for the KIDNET group were 1.76 (CI 0.9–2.5) at post-test and 1.96 (CI 1.1–2.8) at 6-months follow-up. The corresponding effect sizes for MED-RELAX were 1.83 (CI 0.9–2.6) and 2.20 (CI 1.2–3.0).

### PTSD diagnosis

6 months after treatment, 81% of the children in the KIDNET group and 71% of those treated with MED-RELAX did not reach the PTSD -RI threshold for a PTSD diagnosis. Results of this analysis are presented in Table 3. Differences between groups were non-significant at both time-points as confirmed by χ^2 ^tests.

**Table 3:** Number and percentage of children with a diagnosis of PTSD according to the UPID at posttest and 6-months follow up.

	1 month post test		6-months follow up	
	PTSD	No PTSD	PTSD	No PTSD

NET	4 (25%)	12 (75%)	3 (18.7%)	13 (81.3%)
MED-RELAX	5 (33.3%)	10 (66.6%)	4 (28.6%)	10 (71.4%)

Note. χ^2 ^tests did not show any significant group differences

### Functional impairment and physical problems

In addition to PTSD symptoms, problems in different areas of daily functioning (school performance, social relationships, family etc.), as well as physical complaints, were assessed in children before and after treatment (see table 4 for values). For each outcome measure (number of physical complaints, number of functioning problems), a repeated measures ANOVA was calculated with Time as a three-level within-subject variable and Treatment as a two-level between subjects variable. With respect to impairments in functioning, the ANOVA resulted in a significant main effect Time (F(2,54) = 19.54; p < 0.001) with no significant Time × Treatment interaction. Across both conditions, a slight improvement for physical problems was visible after treatment. However, the Time effect did not reach significance (F (2,56) = 2.47; p = 0.09).

**Table 4:** Means and standard deviations of number of functioning problems and physical symptoms of the two treatment groups at pre-test, post-test, and follow up time points.

	pre-test	post-test	6 -months follow-up
Functioning problems			
KIDNET	2.06 (1.34)	0.50 (0.82)	0.44 (0.81)
MED-RELAX	2.14 (1.17)	0.80 (0.94)	0.71 (0.99)
Number of physical symptoms			
KIDNET	1.75 (1.34)	1.50 (1.55)	1.50 (1.41)
MED-RELAX	1.80 (1.26)	0.67 (0.62)	1.29 (1.14)

Note. No significant group differences as confirmed by the ANOVA

## Discussion

In the context of the immediate aftermath of a mass disaster, we wanted to test the efficacy of two pragmatic short-term interventions with respect to reduction of PTSD symptoms and functional impairment in traumatized children. We compared a meditation-relaxation protocol with KIDNET, both approaches being carried out by former schoolteachers who had been trained as counselors. Results showed that in both treatment conditions, PTSD symptoms were significantly reduced at 1 month post-test and remained stable over time. At 6 months after completion of therapy, recovery rates were 81% for the children in the KIDNET group and 71% for those in the MED-RELAX group. However, as we could not control for spontaneous remission in this study, these effects have to be evaluated in the context of other longitudinal studies that did not apply any intervention.

A recent study with child victims of accidents in London has found a 50% rate of spontaneous recovery in the first six months [24]. The same rate was found among children who were traumatized by Hurricane Andrew in Florida in the first 10 months after the disaster [31]. However, it is questionable whether these recovery rates can be transferred to the war-torn Tamil school children. On average, the Tamil children had experienced three distinctly different types of traumatic events in addition to the Tsunami, and nearly 70% of the children in the present sample were traumatized by repeated war-related experiences. In fact, within this study, not all of the children did consider the Tsunami as the worst experience, and high levels of lasting mental disorders, including PTSD, had been found among war-affected Tamil school children already before the Tsunami [28]. Retrospective [32] and longitudinal [33] studies revealed that children's preexisting levels of anxiety and psychological maladjustment are among the main predictors of an adverse trajectory of the disorder. Therefore, one might speculate that spontaneous recovery rates would be below the 50% found in the studies in safer regions. In addition to these pre-trauma factors, it is reasonable to assume that the context of a humanitarian emergency after a mass disaster in a country with limited resources has probably also interfered with recovery, in particular because political violence in the North-East of Sri Lanka had begun to rise soon after the Tsunami. Related to this, a survey conducted one year after the Tsunami in the North-East of Sri Lanka showed a PTSD prevalence of 30.4% in a sample of school children out of which 30% had not even been affected by the Tsunami [29] but 49% reported war experiences that had occurred in the past year.

This evidence taken together leads to the conclusion that the recovery rates of 70% and 80% that remained stable over a 6 months post treatment period seem to be higher than the percentages that could be expected from natural recovery in this specific population, and that there is good reason to assume that both interventions have been effective in decreasing PTSD. This assumption is also supported by comparing the rates of 19% and 29% of children in the present study who were diagnosed with PTSD six months after the interventions to prevalence rates of PTSD at six months following natural disasters in resource-poor countries. In this regard, Goenjian and colleagues [2] who investigated the severity of posttraumatic stress among Nicaraguan adolescents after Hurricane Mitch reported estimates of PTSD of 55% to 90% in two very affected cities of the countries six months after the natural disaster had occurred.

In addition to the reduction of psychopathology, we also found a decrease in impairments in psychosocial functioning. This result is of importance since it indicates that both interventions might have had a significant effect on the ability of children to function in their daily lives, e.g. regarding their social relationships and their everyday tasks. We could also show, at least on a statistical trend level, that physical health improved through both therapeutic approaches. This is in agreement with a previous study using NET with an adult refugee population, where an improvement of physical symptoms was found even several months after the intervention [34].

We did not find any significant difference between the KIDNET and the meditation-relaxation protocol groups in any outcome measure. Effect sizes at post-test and 6-month follow up were similarly high in both groups. However, this finding was probably not only determined by a lack of power caused by small sample size. Rather, our findings indicate that, in the immediate aftermath of a disaster, KIDNET is not more efficient than a relaxation-mediation protocol developed by local counselors. This result is in contrast with research on adult trauma treatment that shows a clear advantage of trauma-focused procedures that contain trauma exposure [35], also in the immediate aftermath of the event.

It could be speculated that in the immediate aftermath of a mass disaster, the affected children are unavoidably confronted with trauma reminders in everyday life even in the absence of a systematic exposure intervention. In such a context, strategies that may support fear extinction, like meditation [36], could be similarly effective as exposure therapy. Also, when evaluating an adequate mental health intervention strategy, the cultural applicability of the therapy approach has to be taken into account. With respect to the Sri Lankan context, meditation and relaxation exercises can be seen as part of the Tamil culture. Children, as well as their teachers, are well used to relaxation techniques, such as breathing exercises, mantra chanting or meditation. These are common activities in the classroom. Further clinical studies in other countries of different cultures are needed to verify whether relaxation-mediation techniques are effective in treating PTSD in children regardless of the cultural background of the affected community. In contrast, the efficacy of NET has already been shown across cultures in Europe, Africa and Asia [15,16,18].

With this study we could also show that local counselors with a relatively short training are able to carry out psychological interventions, including trauma exposure and meditation-relaxation. Particularly in the context of large-scale traumatization, the use of local personnel with no specific background in trauma treatment is of high significance to the development of efficient ways in dealing with the epidemic rates of posttraumatic stress disorder.

The major limitation of this study is the lack of a non-treated control group to control for spontaneous remission. Given the urgent request for trauma interventions among the population in the camp where the study was conducted, we decided not to include such a group because of ethical concerns. As stated before, because of the insecure and temporary shelter in the aftermath of the Tsunami disaster, we could not be sure, whether it would have been possible at all to relocate children assigned to a waiting list condition again for later treatment. However, as outlined above, even taking into account natural recovery rates, we have reason to conclude that the PTSD symptom remission obtained in the present trial supports the beneficial effect of both, KIDNET and a meditation-relaxation protocol applied in the immediate aftermath of a natural disaster.

Given the increasing violence and political insecurity in Sri Lanka's North-Eastern provinces during the year after the Tsunami disaster, the follow-up interviews that were planned for 1 year after the completion of therapies could not be carried out. Diagnostic interviews at a later stage could have provided a clearer insight into therapy effects, in particular with respect to NET. Previous studies with adolescents and adults have shown that PTSD symptom reduction at one year follow-up is more pronounced than effects at three to four-months post-tests when administering NET [16,17].

## Conclusion

In conclusion, we can state that this study provides preliminary support for the effectiveness of a KIDNET as well as a meditation-relaxation protocol when carried out with traumatized children in the acute stage of mass disaster. In the future, these findings need to be replicated in other cultural settings, preferentially with larger samples, an untreated control group and additional outcome measures.

## Competing interests

The authors declare that they have no competing interests.

## Authors' contributions

CC developed the design, trained the local counselors, supervised therapies, supervised the post-treatment assessments, performed the statistical analysis and drafted the manuscript. MK trained and supervised the local counselors, supervised therapies, and organized and supervised post-treatment assessments. MR developed the design, trained the local counselors and supervised therapies. ES trained the local counselors and participated in initial coordination of the survey. TE conceived of the study and participated in its design. FN participated in study design, training of the counselors, and manuscript preparation. All authors read and approved the final manuscript.

## Pre-publication history

The pre-publication history for this paper can be accessed here:


